# The immunity protection of intestine induced by pseudorabies virus del gI/gE/TK in piglets

**DOI:** 10.3389/fmicb.2023.1295524

**Published:** 2024-01-05

**Authors:** Yang Zhang, Lei Xu, Qian Tao, Zheyan Liu, Jianhua Wen, Tong Xu, Siyuan Lai, Yanru Ai, Zhiwen Xu, Ling Zhu

**Affiliations:** ^1^College of Veterinary Medicine, Sichuan Agricultural University, Chengdu, China; ^2^Key Laboratory of Animal Disease and Human Health of Sichuan Province, Chengdu, China

**Keywords:** pseudorabies virus, intestinal damage, intestinal immunity, inflammation, intestinal barrier

## Abstract

Compared to the classical strain of Pseudorabies virus (PRV), the PRV variant exhibits stronger transmissibility and pathogenicity, causing immense disasters for the global pig industry. Based on this variant, our laboratory has preliminarily constructed a modified pseudorabies virus with deletions in the gE/gI/TK genes. In this study, the protective efficacy of PRV XJ del gI/gE/TK against piglet intestinal damage was evaluated. The results demonstrated that piglets immunized with PRV XJ del gI/gE/TK exhibited alleviated intestinal damage caused by the PRV XJ variant strain. This included reduced viral load, suppressed inflammation, and maintenance of intestinal structure and function. Additionally, PRV XJ del gI/gE/TK also strongly activated the innate immune response in the intestines, increasing the expression of antiviral factor mRNA and the secretion of SIgA to counteract the attack of the PRV XJ variant strain. Our study indicates that PRV XJ del gI/gE/TK can inhibit intestinal damage caused by PRV XJ variant strain and activate the innate immune response in the intestines.

## 1 Introduction

Pseudorabies virus, also known as Aujeszky virus, belongs to the *Herpesviridae* family, a member of the *Alphaherpesviridae* subfamily, and has the ability to infect a wide range of mammals, including pigs, cattle, sheep, and dogs. However, pigs are the only natural host for this virus (Deng et al., 2022; Liu et al., 2022). Since 2011, a severe pseudorabies epidemic has occurred in pig farms where the animals were immunized with the Bartha-K61 vaccine (An et al., 2013). This outbreak has resulted in significant economic losses for the pig industry. Analysis of the viral genome has revealed that the newly emerged PRV variant belongs to a strain variant of genotype 2. In recent years, extensive serological molecular epidemiological studies on PRV infection have been conducted in pig populations in China to better control the spread of the novel variant strains (Zhao et al., 2023). The prevalence of PRV infection is influenced by various factors such as region, sample size, and season. According to a survey, the average serum positivity rate for PRV gE was found to be 29.87% in 256,326 serum samples from different regions of 29 provinces in China. The seroprevalence in North China, East China, Central China, and South China was higher than that in Northeast, Northwest, and Southwest China (Tan et al., 2021). Furthermore, another investigation revealed that, since 2011, the majority of isolated PRV strains belong to the II genotype, with only a few being members of the I genotype. This indicates that the II genotype variant strains of PRV are more prevalent in China (Zhai et al., 2019).

It is widely believed that severe neurological symptoms and systemic inflammation are the main causes of acute mortality in hosts infected with PRV (Zheng et al., 2022). Upon entering the host, PRV initially replicates in the epithelial cells of the upper respiratory tract. Subsequently, it traverses the cell basement membrane to access peripheral nerve endings and the bloodstream. Through the peripheral nervous system and circulatory system, PRV can infect the central nervous system and various organs throughout the body. Ultimately, PRV can activate multiple inflammatory pathways, inducing lethal inflammatory responses both internally and externally, ultimately leading to host death (Ye et al., 2022). Studies have demonstrated that PRV infection can lead to necrotizing enteritis and disruption of the intestinal flora, ultimately causing a disturbance in intestinal homeostasis (Narita et al., 1984; Ezura et al., 1995; An et al., 2013). Changes in the intestinal environment can further exacerbate the disruption of the flora, leading to increased inflammation. These findings underscore the detrimental effects of PRV on the intestinal tract of piglets.

The intestinal mucosal barrier plays a crucial role as the first line of defense against external harmful factors and in maintaining intestinal homeostasis (Chen et al., 2021). Its integrity primarily relies on epithelial cells and tight intercellular connections that effectively prevent the invasion of pathogens and endotoxins into the bloodstream through the intestinal mucosa (Perrin and Matic Vignjevic, 2023). Additionally, the chemical barrier formed by the mucous secreted by goblet cells contains a variety of anti-secretory factors, such as secretory immunoglobulin A (SIgA) and bacteriostatic peptides, which further reinforce the protective function of the mucosal barrier beyond the epithelium (Luis and Hansson, 2023). Consequently, the intestinal mucosal barrier plays a significant role in the innate immune response of the host.

This study aims to assess the protective effects of the previously developed gE/gI/TK-deficient PRV in piglets, with a particular focus on the intestinal aspects. The vaccine demonstrated promising results in preliminary studies conducted in mice, effectively shielding them from the lethal assault of the PRV XJ variant strain. It inhibited the neural damage and inflammation induced by the PRV XJ variant strain, activating innate immunity in the brain and intestinal tract (Xu et al., 2022). The results of this study showed that PRV XJ del gI/gE/TK vaccine effectively protects piglets from PRV XJ variant strain by activating intestinal immunity and has a strong protective effect.

## 2 Materials and methods

### 2.1 Virus and cells

The PRV XJ strain (GenBank accession no. MW893682), identified as a variant strain, was isolated and characterized by our laboratory. Subsequently, our laboratory successfully constructed a PRV XJ del gI/gE/TK variant, in which the gI, gE, and TK genes were deleted (Xu et al., 2022). To propagate all viruses, the PK-15 cells were cultured in Dulbecco’s Modified Eagle’s Medium (DMEM) supplemented with 10% (v/v) fetal bovine serum.

### 2.2 Animal and experiment design

Landrace piglets, approximately 5 days old, were employed as subjects in this study. Prior to the experiment, all piglets were tested negative for antibodies against PRV gB (ID-VET, French) and gI (IDEXX, Westbrook, ME, USA). Furthermore, they were confirmed to be free of porcine reproductive and respiratory syndrome virus (IDEXX, Westbrook, ME, USA), porcine circovirus 2 (Zhenrui Bio, Shenzhen, China), porcine epidemic diarrhea virus (IDEXX, Westbrook, ME, USA), and classical swine fever virus (IDEXX, Westbrook, ME, USA).

Nine piglets were randomly assigned to three groups: the PRV XJ-infection group (Vehicle), the PRV XJ del gI/gE/TK-immunization group (Vaccination), and the Mock group (*n* = 3 in each group). Throughout the study, the piglets had *ad libitum* access to food and water and were housed at room temperature (23 ± 1.5°C). The piglets in the immunization group were intranasally inoculated with 10^7^ TCID_50_ of PRV XJ del gI/gE/TK exhibited 100% protective efficacy (Jun, 2023). Furthermore, booster vaccinations were performed at week 2. The piglets in the vehicle group and control group received intranasal inoculations of DMEM. At week 3 following the vaccinations, the piglets in the immunization and infection groups were challenged with 10^6^ TCID_50_ of PRV XJ strain via intranasal inoculation. The piglets in the control group were intranasally inoculated with DMEM. Previous studies have demonstrated that high levels of PRV DNA can be detected in the nasal mucosa, lung, spleen, liver, and brain of 2-week-old piglets at day 5 post-infection, as well as in 15-week-old pigs at day 5 post-infection. Additionally, extensive viral replication accompanied by robust expression of cytokine mRNA was observed in the brain of pigs (Verpoest et al., 2017). Therefore, on day 5 after the challenge, all piglets were euthanized by intravenous injection of pentobarbital sodium (100 mg/kg). Throughout the study, the piglets were monitored daily for body temperature, clinical signs, and virus shedding following the challenge.

### 2.3 Test for serological antibodies

Blood samples were collected at 1, 4, 7, 10, 13, 14, 16, 19, 21, 22, and 25 days post- immunization (dpi). After coagulation, the serum was separated through centrifugation, and the presence of PRV-specific gB and gI antibodies in the serum was assessed using ELISA kits as per the manufacturer’s instructions. For gB-specific antibodies, a positive result is defined as S/N ≤ 0.3, suspicious is 0.3 < S/N ≤ 0.4, and negative is S/N > 0.4. For gI-specific antibodies, a positive result is S/N ≤ 0.6, suspicious is 0.6 < S/N ≤ 0.7, and negative is S/N > 0.7. In addition, neutralization assays were performed on serum samples collected 14 days and 21 days after immunization. In this assay, 50 μL of serum samples were serially two-fold diluted and incubated with PRV-XJ strain at a concentration of 100 TCID_50_ of the PRV XJ strain at 37°C for 60 min. The mixture was then added to confluent PK-15 cells cultured in 6-well plates and incubated at 37°C, 5% CO_2_ for 2–3 days. Cells were then harvested to determine cytopathic effect (CPE) and plaque counts. Neutralizing antibody titers were calculated by the Reed-Muench method.

### 2.4 Histopathology analyses

Tissue samples obtained from segments of the duodenum, jejunum, ileum, and colon of piglets were fixed in 4% paraformaldehyde for a minimum of 72 h. Paraffin sections were prepared using a series of steps including dehydration, clearance, embedding, and sectioning. To visualize any pathological changes in the intestinal tissue samples, the hematoxylin and eosin (H&E) staining protocol (Solarbio, Beijing, China) was employed. Villous height and crypt depth were assessed by measuring a minimum of 5 villi and crypts across the section. The mean ratios of intestinal villous height to crypt depth (VH:CD) were calculated following previously described methods (Jung et al., 2006; Zhao et al., 2021).

### 2.5 Alcian blue periodic acid Schiff staining and immunohistochemistry

Paraffin-embedded colon specimens with a thickness of 4 μm were subjected to Alcian blue periodic acid Schiff (AB-PAS) staining to analyze the presence of goblet cells. The samples were stained using an Alcian Blue staining solution, followed by treatment with 1% aqueous periodic acid and staining with Schiff’s reagent. Nuclei were lightly stained with hematoxylin. Afterward, the samples were differentiated using acid alcohol, washed with Scott’s tap water, and then dehydrated, cleared, and mounted on glass slides with coverslips. The number of goblet cells was determined by counting the Alcian Blue-positive vacuoles under a light microscope.

Paraffin-embedded intestinal tissue samples with a thickness of 4 μm were subjected to immunohistochemistry to analyze the presence of SIgA. After antigen retrieval, the samples were incubated overnight at 4°C with SIgA antibodies (ab112746, Abcam, diluted 1:500). Subsequently, the samples were stained with 3,3′-diaminobenzidine and counterstained with hematoxylin. Finally, the specimens were dehydrated, cleared, and mounted on glass slides with coverslips. The average optical density of intestinal SIgA was quantified using ImageJ software.

### 2.6 Quantitative real-time PCR assay

The fresh colonic intestines of piglets were dissected to assess the expression of inflammation factors and antiviral factors in the intestine. RNA extraction from the piglet’s intestine was performed using RNAiso Plus following the manufacturer’s protocols. The concentration and purity of the extracted RNA were determined using ScanDrop, measuring the A260 value and the A260/280 ratio, respectively. Reverse transcription reactions were carried out using the PrimeScript RT Kit. Quantitative RT-PCR was performed on a Roche Lightcycler96 instrument using the TB Green Premix Ex Taq in accordance with the manufacturer’s instructions. The forward and reverse primer sequences for each gene are provided in Table 1. Gene expression levels were quantified using the 2^^–ΔΔCT^ method.

**Table 1:** Primers for qRT-PCR.

Primer name	Sequence	Size	Cite
gE-F	CTTCCACTCGCAGCTCTTCT	165 bp	Xu et al. (2022)
gE-R	TAGATGCAGGGCTCGTACAC		
PRV IE180-F	TAAGTCCGGCTACAGCACCAAGTCC	207 bp	Tao et al. (2023)
PRV IE180-R	TCTTCGTCGTCGCGGTGGGGCCGT		
IFN-β-F	ACCAACAAAGGAGCAG	222 bp	Deng et al. (2022)
IFN-β-R	TTTCATTCCAGCCAGT		
IFN-γ-F	AACCAGGCCATTCAAAGGAGC	149 bp	
IFN-γ-R	TCACTGATGGCTTTGCGCTG		
ISG15-F	ATGTGCTTCAGGATGGGGT	99 bp	
ISG15-R	GGATGCTCAGTGGGTCTCT		
OAS1-F	ACCTCGATAACATGCTGGAC	162 bp	
OAS1-R	TGATGGTGAAAGTGATGGGC		
IL-1β-F	AACCAAGCAACGACAAAATAC	131 bp	
IL-1β-R	CTTCTTTGGGTATTGCTTGGG		
IL-6-F	ATGAACTCCCTCTCCACAAGC	119 bp	
IL-6-R	GCATCACCTTTGGCATCTTCT		
IL-8-F	CAACAAGCAAAAACCCATTC	132 bp	
IL-8-R	CTGTGATTTCTCTGGCAAC		
IL-10-F	ATCCACTTCCCAACCAGCCTG	138 bp	
IL-10-R	ACCCTTAAAGTCCTCCAGCAG		
TNF-α-F	TCAGCCTCTTCTCCTTCCTCC	168 bp	
TNF-α-R	CGACGGGCTTATCTGAGGTTT		
β-actin-F	GCTGTGCTATGTTGCTCTAG	179 bp	Deng et al. (2022)
β-actin-R	CGCTCGTTGCCAATAGTG		

Total DNA was extracted from various tissues of the piglets using a universal genomic DNA kit. The viral loads in tissue samples were assessed using the qRT-PCR assay targeting the PRV gE gene. The gene copy number for each sample was expressed as log10 copies per gram of tissue sample.

### 2.7 Enzyme-linked immunosorbent assay

After euthanizing the piglets, blood samples were collected. The serum was separated by centrifugation following coagulation, and the serum was subjected to detection of IL-1β (IDEXX, Bern, Switzerland), IL-4 (IDEXX, Bern, Switzerland), IL-6 (IDEXX, Bern, Switzerland), IL-8 (IDEXX, Bern, Switzerland), IL-10 (IDEXX, Bern, Switzerland), TNF-α (IDEXX, Bern, Switzerland), and DAO (Neobioscience, China) using ELISA kits. The optical density at 450 nm was measured using a microplate reader (Bio-Rad, Hercules, CA, United States).

### 2.8 Western blotting assay

Intestinal proteins were extracted and homogenized using lysis buffer containing the protease inhibitor phenylmethanesulfonyl fluoride. The protein concentration was determined using the BCA Protein Assay Kit. Equal amounts of total protein were then separated by sodium dodecyl sulfate-polyacrylamide gel electrophoresis and transferred onto a polyvinylidene fluoride membrane. The membrane blots were saturated with 5% BSA in phosphate-buffered saline with Tween 20 (PBST) for 2 h at room temperature, followed by overnight incubation at 4°C with primary antibodies against ZO-1 (21773-1-AP, Proteintech, diluted 1:5000), Occludin (27260–1, Proteintech, diluted 1:1000), and β-actin (AC026, Abclonal, diluted 1:50,000). After incubation, the membrane was washed three times with PBST and then incubated with HRP-conjugated Goat Anti-Rabbit IgG (H + L) (AS014, Abclonal, diluted 1:10,000). The signals were visualized using the SuperSignal™ West Pico Plus Chemiluminescent Substrate. The gray intensity of the proteins was measured using ImageJ software.

### 2.9 Data analysis

Statistical analysis was undertaken by one-way analysis of variance with GraphPad 7.04 software. All results were expressed as mean ± standard deviation from at least three replicates and were representative of three independent experiments. The value of *p* < 0.05 was considered statistically significant.

## 3 Results

### 3.1 Humoral immunity response induced by PRV XJ Del gE/gI/TK in piglets

**Figure 1:**
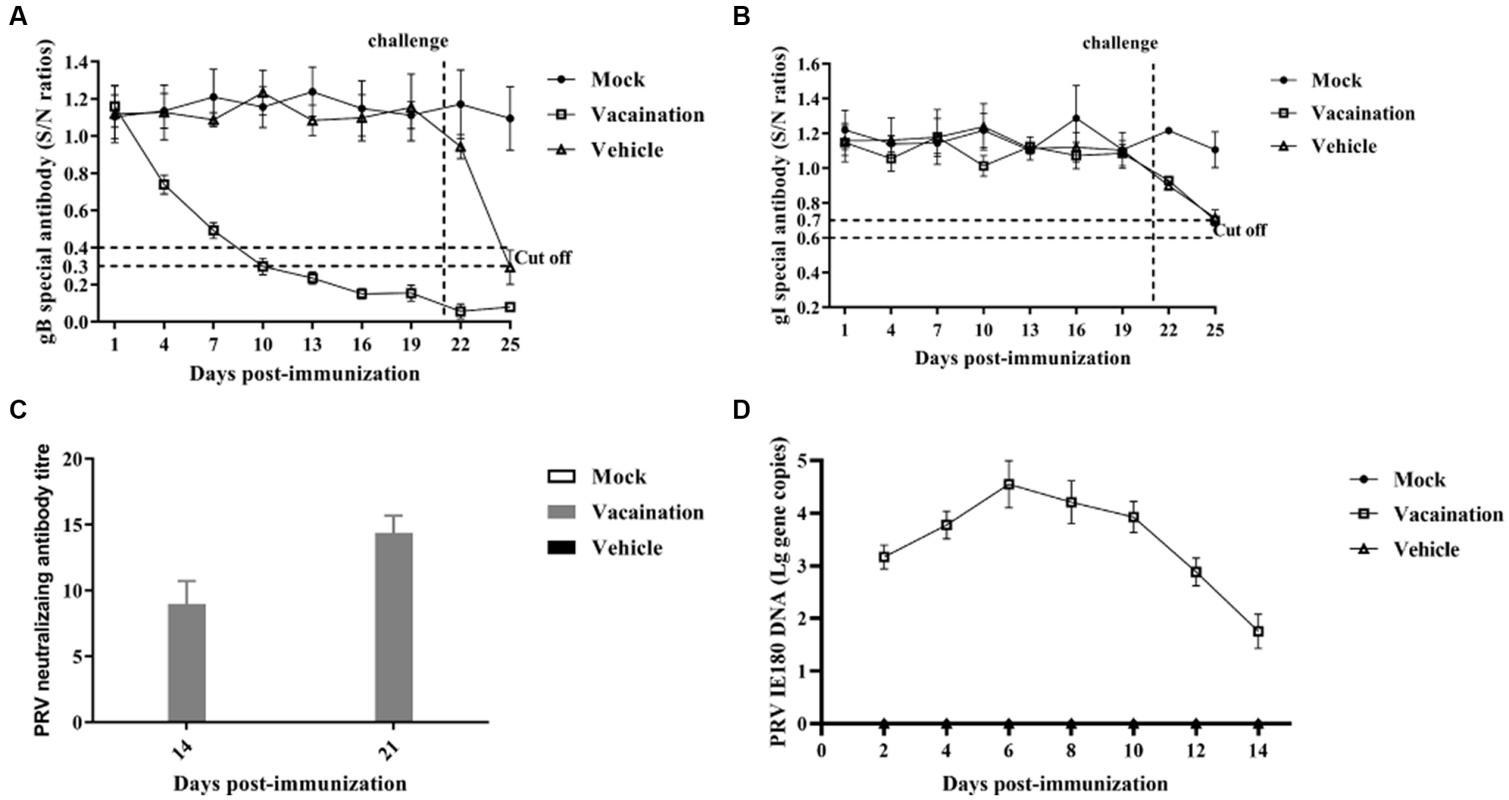
Immune responses after immunization with PRV XJ del gI/gE/TK. **(A)** Calculation of gB-specific antibodies using ELISA. **(B)** Calculation of gI-specific antibodies using ELISA. **(C)** Neutralizing antibody titers against the PRV XJ. **(D)** PRV IE180 copies in nasal swabs was detected by qPCR.

To assess the specific antibody response induced by PRV XJ Del gE/gI/TK, to assess the specific antibody response induced by PRV XJ Del gE/gI/TK, indirect ELISA assays were utilized to detect antigen-specific antibodies and a specific virus-neutralizing antibody (VNA) test were utilized to detect neutralizing antibodies. The antibodies to gB turned positive at 10 days post-immunization (dpi), and their levels gradually increased over time after vaccination. And the antibody levels of gB plateau between 19 and 22 days in vaccination group. Before the PRV XJ strain challenge, gB-specific antibodies and gI-specific antibodies were not detected in the mock and vehicle groups (Figures 1A,B). However, after the PRV XJ strain challenge, gI-specific antibodies in both the vaccination group and the vehicle group turned the suspected positive at 5 days post-challenge (dpc). Meanwhile, there were no differences among the vaccination and vehicle groups. The serum samples were further evaluated for their ability to neutralize PRV through a neutralizing test (Figure 1C). The neutralization activity against the PRV-XJ strains was assessed using a plaque reduction assay in the vaccination group before the challenge. The neutralizing antibody titers ranged from 1:6.92 to 10.07 at 14 dpi and from 1:13.16 to 15.74 at 21 dpi (Figure 1C). The shedding of the PRV XJ Del gE/gI/TK virus in piglets was evaluated using qPCR. Following intranasal immunization with 10^7^ TCID_50_ of PRV XJ Del gE/gI/TK the PRV IE180 gene copies peaked at 6 dpi, and gradually decreased (Figure 1D). These findings indicate that PRV XJ Del gE/gI/TK induces an immune response in piglets, enabling them to defend against PRV-XJ challenges.

### 3.2 PRV XJ Del gE/gI/TK protects piglets against variant PRV XJ strain

**Figure 2:**
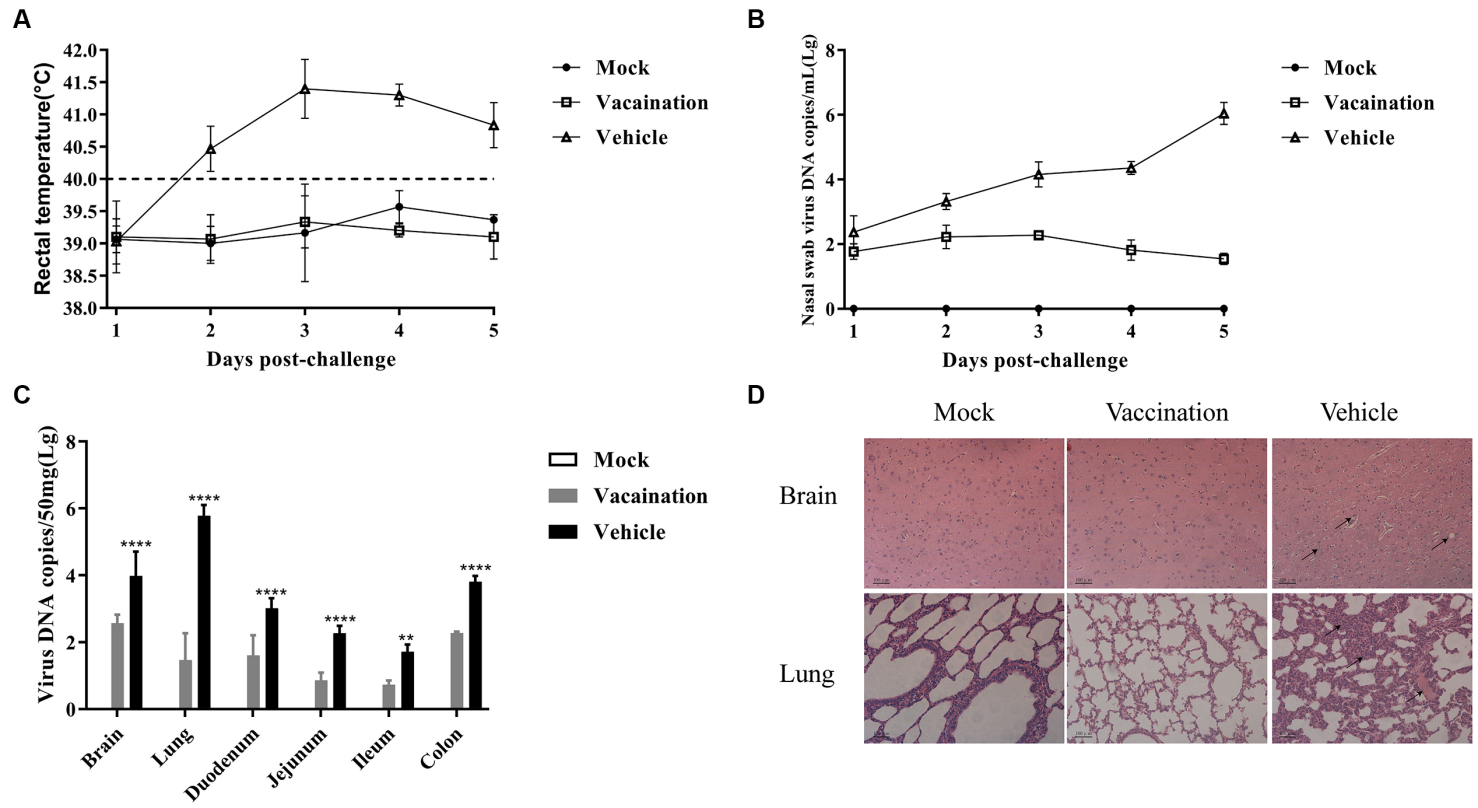
Protective effects of PRV XJ del gI/gE/TK on piglets. **(A)** After PRV XJ challenge, rectal temperatures of piglets in each group were monitored, with rectal temperature > 40.5°C considered as fever. **(B)** Viral DNA load in nasal swabs was detected by qPCR. **(C)** Viral DNA load in different tissues was determined by qPCR. **(D)** Histopathological observations of piglet brain and lung tissues infected with PRV XJ, PRV XJ del gI/gE/TK, and DMEM control, stained with H&E. Data are presented as mean ± SD (*n* = 3). *p < 0.05, **p < 0.01, ***p < 0.001, ****p < 0.0001.

The piglets in the vaccination and vehicle groups were intranasally challenged with 10^6^ TCID_50_ of the PRV XJ strain at 21 dpi. The rectal temperatures of all the piglets were monitored. In the vehicle group, all pigs exhibited typical clinical signs such as sneezing, breathlessness, loss of appetite, and dystaxia, accompanied by high fever (>41°C). Additionally, one out of the three piglets in the vehicle group showed significant symptoms of diarrhea. However, the rectal temperatures of all piglets immunized with PRV XJ Del gE/gI/TK remained below 40.0°C following the PRV XJ challenge, and no clinical signs were observed in these piglets (Figure 2A).

The nasal swab samples were collected after the challenge, and the PRV gE copies were determined using qRT-PCR. The vehicle group exhibited higher levels of excreted PRV compared to the vaccination group. Over time, the copy numbers increased. However, the vaccination group demonstrated a peak in PRV shedding at 3 days post-challenge (dpc), followed by a subsequent decline. These findings suggest that PRV XJ Del gE/gI/TK can partially inhibit the shedding of PRV XJ from the nasal cavity (Figure 2B).

At 5 dpc, all piglets were euthanized, and viral loads in different tissues were assessed using qRT-PCR. The vaccination group exhibited lower viral loads in all tissue samples compared to the vehicle group (Figure 2C). The viral genome copies were detected in the brain, lungs, duodenum, jejunum, ileum, and colon of both the vaccination and vehicle groups. As mentioned earlier, the brain and lungs showed the highest viral loads in both groups (Zhou et al., 2022). Additionally, the colon exhibited the highest viral load among the intestinal tissues in both the vaccination and vehicle groups.

The histopathological examination revealed specific findings in different groups. In the vehicle group, the brain displayed vacuolar neuronal degeneration, neuron phagocytosis, and nuclear cleavage. The lungs exhibited congestion, lymphocyte infiltration, and thickening of alveolar septa. In contrast, no histopathological changes were observed in the vaccination group or the mock group.

### 3.3 PRV XJ Del gE/gI/TK inhibited the inflammatory response induced by PRV XJ

**Figure 3:**
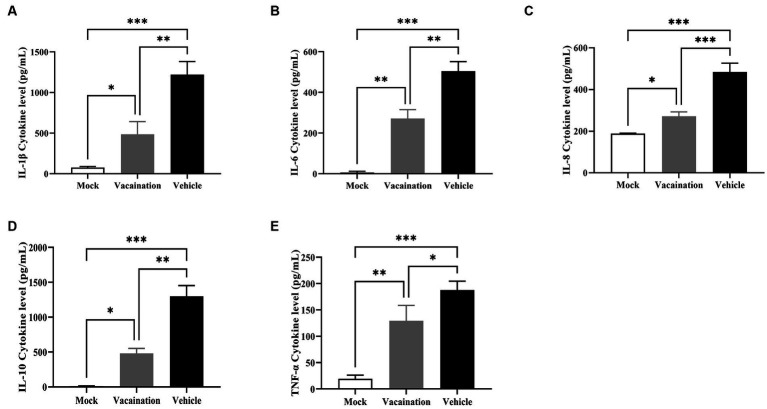
Cellular immune response analysis of Vehicle, Vaccination, and Mock groups. **(A–E)** Represent ELISA assays to examine IL-1β, IL-6, IL-8, IL-10, and TNF-α levels in serum, respectively. Data are presented as mean ± SD (*n* = 3). *p < 0.05, **p < 0.01, ***p < 0.001, ****p < 0.0001.

The main cause of piglet mortality induced by PRV was a systemic inflammatory storm. Hence, the serum levels of inflammatory cytokines were measured in all piglets. It was evident that PRV XJ infection induced an inflammatory response, significantly increasing the levels of IL-1β, IL-6, IL-8, IL-10, and TNF-α in both the vaccination and vehicle groups. However, compared to the vaccination group, the vehicle group exhibited significantly higher levels of IL-1β, IL-6, IL-8, IL-10 and TNF-α (Figure 3). These results collectively indicated that PRV XJ Del gE/gI/TK could suppress the inflammatory response triggered by PRV XJ infection.

### 3.4 Gut integrity is maintained in vaccinates

**Figure 4:**
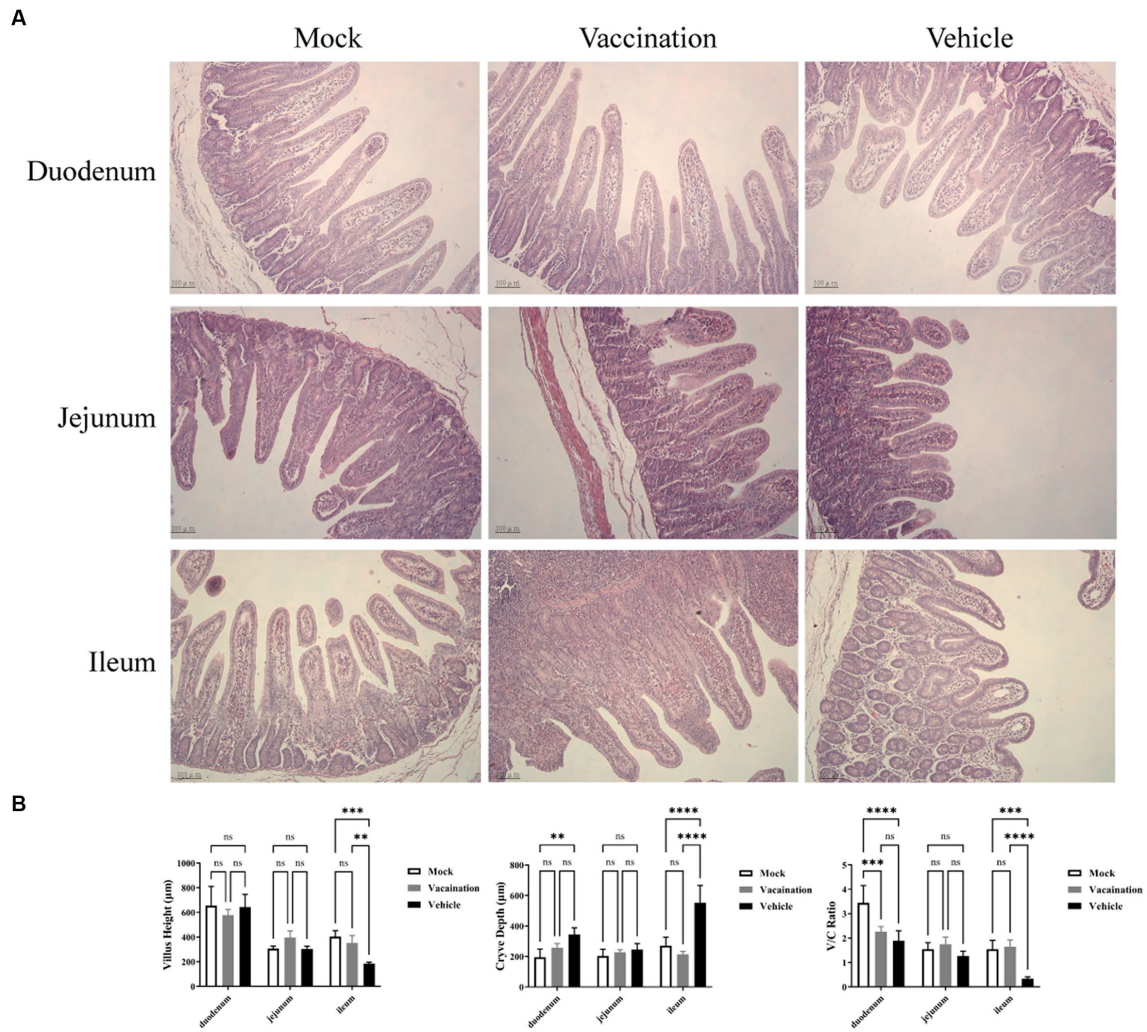
Histopathological alterations in small intestinal tissues. **(A)** H&E stained pathological observations of duodenum, jejunum, and ileum samples collected from piglets of different groups. **(B)** Average intestinal villus height, crypt depth, and their ratio in piglets from different groups. Data are presented as mean ± SD (*n* = 3). *p < 0.05, **p < 0.01, ***p < 0.001, ****p < 0.0001.

To assess the integrity of the intestinal tissues collected at 5 dpc, we measured villus height, crypt depth, and the ratio of villus height to crypt depth in the duodenum, jejunum, and ileum (Figure 4). Among the segments of the small intestine, the ileum exhibited more severe pathological symptoms in the vehicle group, characterized by significant atrophy of intestinal villi and crypt hyperplasia. In comparison to the mock group and the vaccination group, PRV infection led to a considerable reduction in villus height and a marked increase in crypt depth in the ileum of the vehicle group, resulting in a decreased ratio of villus height to crypt depth. However, there were no significant differences in villus height and crypt depth observed in the duodenum and jejunum among all groups (Figure 4B).

**Figure 5:**
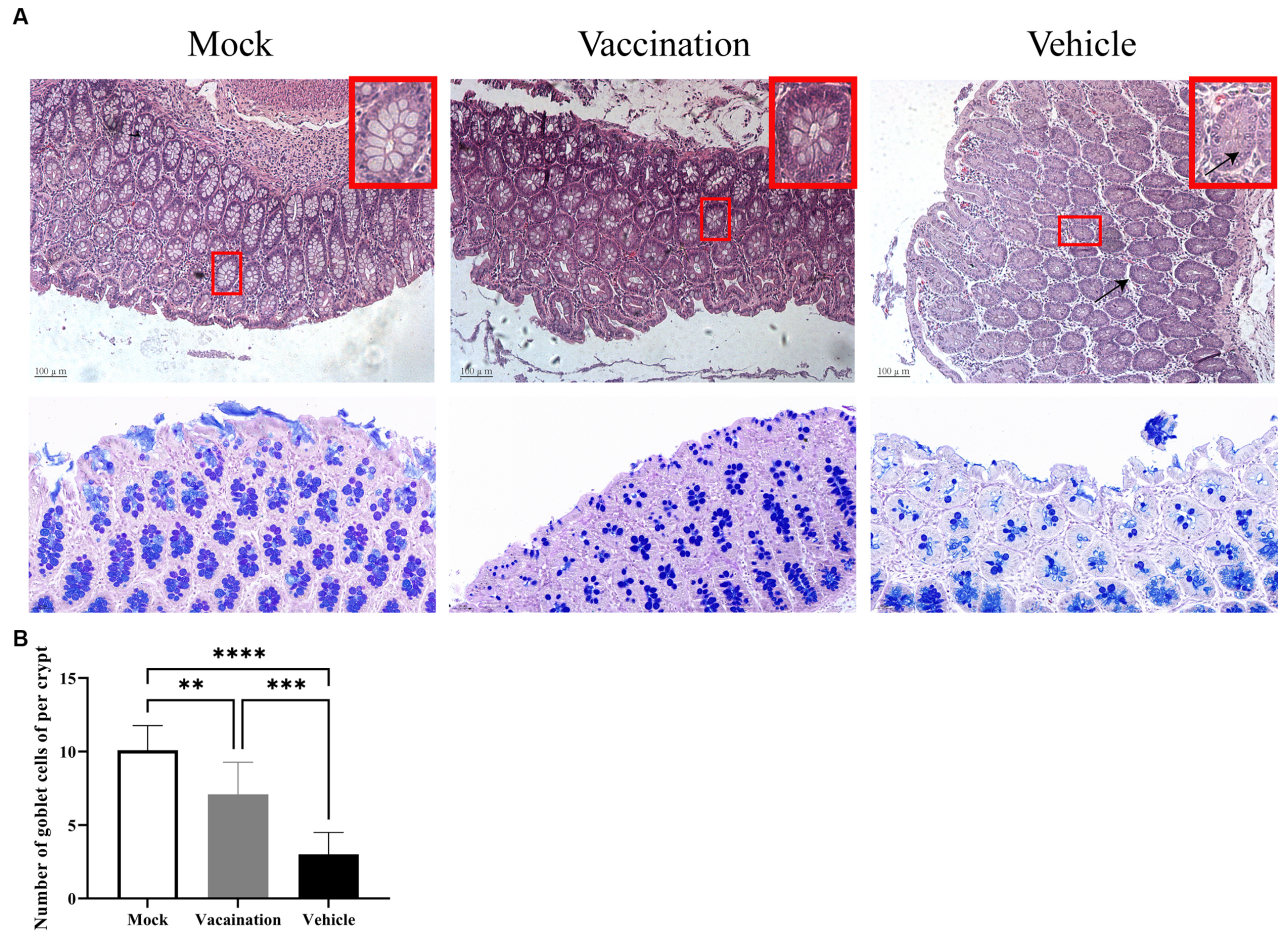
Colonic histopathological alterations. **(A)** H&E stained pathological observations of colonic tissues in piglets from different groups. **(B)** Detection of colonic goblet cell count using AB-PAS staining method. Data are presented as mean ± SD (*n* = 3). *p < 0.05, **p < 0.01, ***p < 0.001, ****p < 0.0001.

Furthermore, the histopathological examination of the colon in the vehicle group revealed a decrease in mucus within the colonic crypts, accompanied by increased spacing between crypts due to edema. Conversely, no histopathological changes were observed in the colon of the vaccination group or the mock group (Figure 5A). Examining the changes in goblet cell number and mucopolysaccharide content is crucial for diagnosing intestinal functional and structural alterations (Chairatana and Nolan, 2017). In AB-PAS staining, acidic mucous substances were stained lake blue by AB staining, while neutral mucous substances were stained purple-blue by PAS staining. As depicted in Figure 5B, AB-PAS staining demonstrated densely distributed, regularly-shaped goblet cells with positive staining for mucous substances on both sides of the crypt in the colonic mucosa of the mock group and vaccination group. In contrast, the vehicle group exhibited exfoliated mucosal layers, few remaining crypts within the lamina, and a noticeable decrease in the proportion of positively stained goblet cells containing mucus.

### 3.5 PRV XJ Del gE/gI/TK prevents the increase of intestinal permeability and the decrease of tight junction expression induced by PRV XJ

**Figure 6:**
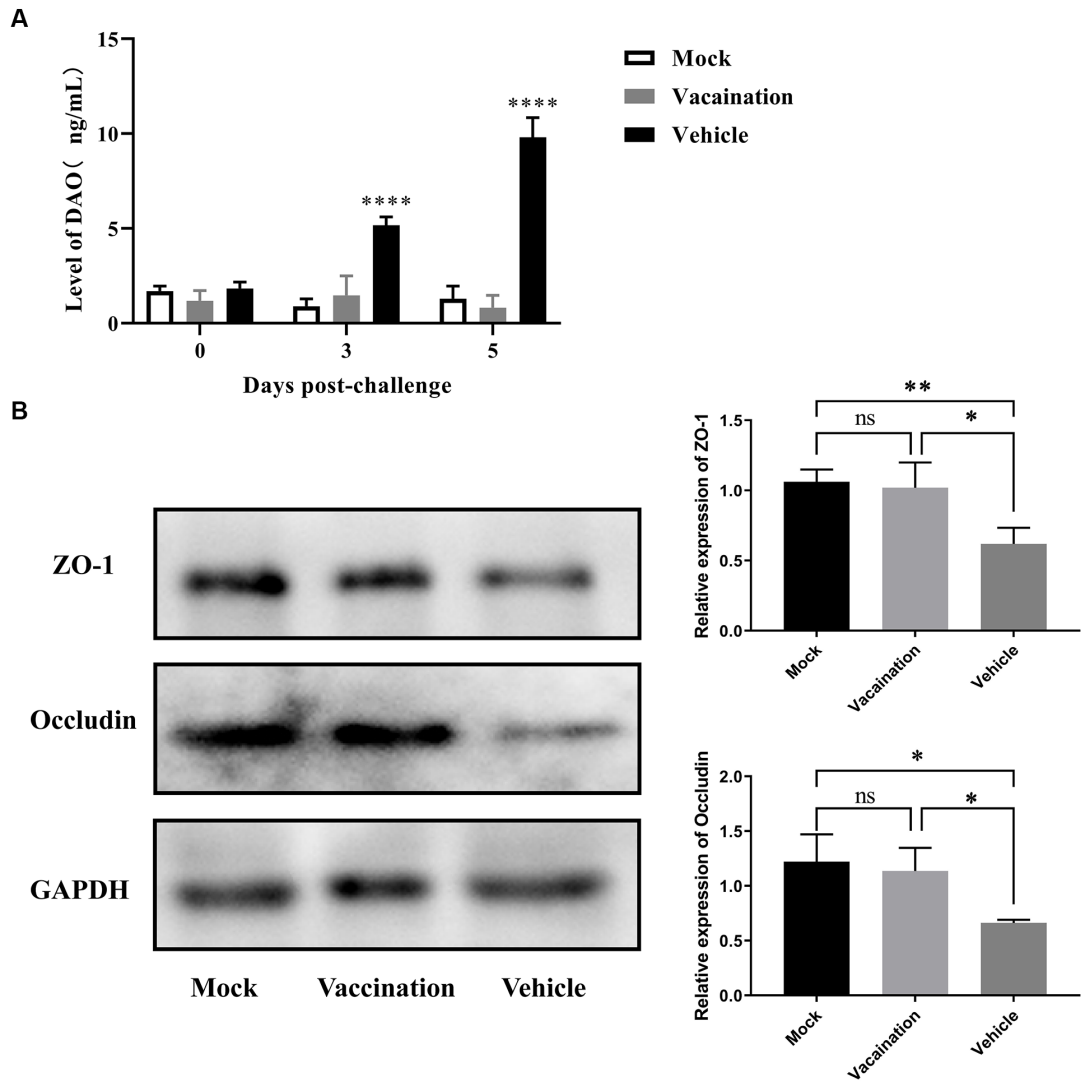
Assessment of intestinal integrity in Vehicle, Vaccination, and Mock groups of piglets. **(A)** Serum diamine oxidase levels were measured using ELISA. **(B)** Western blot analysis and relative quantification of the band density of tight junction(Occludin and ZO-1). Data are presented as mean ± SD (*n* = 3). *p < 0.05, **p < 0.01, ***p < 0.001, ****p < 0.0001.

Intestinal permeability and the function of epithelial tight junction barriers are critical in the development of diarrhea (Vanuytsel et al., 2021). To assess the intestinal barrier function, the serum levels of DAO were measured using an Elisa, and the expression of tight junction proteins in the colon was measured using Western blotting. The results showed a significant increase in serum DAO levels in the vehicle group compared to the mock group and vaccination group (Figure 6A). Additionally, Western blotting revealed a significant reduction in the expression of ZO-1 and occludin in the colon as a result of PRV infection (Figure 6B). However, there were no differences observed in serum DAO levels and the expression of ZO-1 and occludin between the vaccination group and mock group. This suggests that PRV XJ Del gE/gI/TK provided a certain level of protection for the structure and barrier function of the intestinal mucosal epithelium.

### 3.6 PRV XJ Del gE/gI/TK inhibited inflammation and activated strongly immunity in intestine

**Figure 7:**
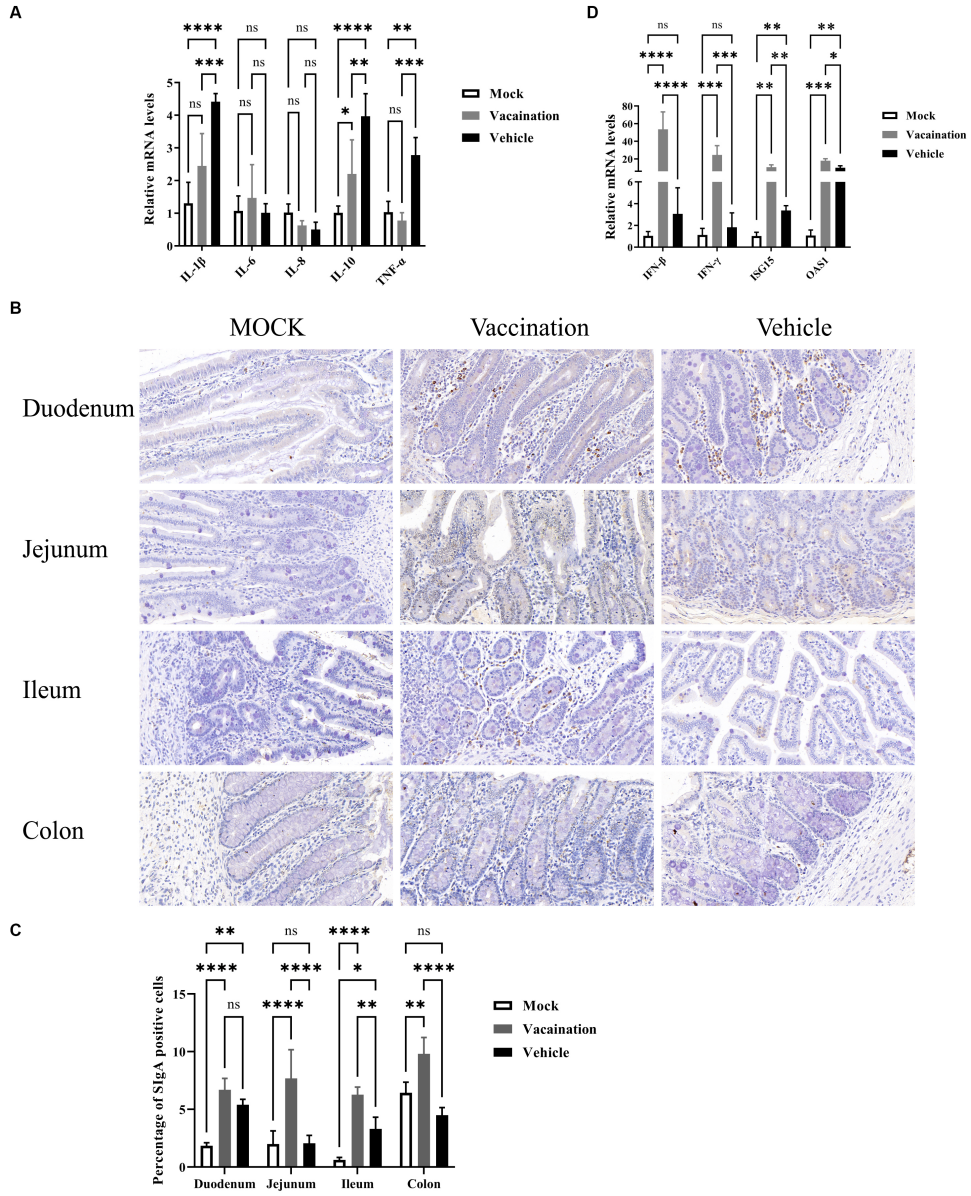
Protective role of PRV XJ del gI/gE/TK in piglet intestines. **(A)** The fold change of IL-1β, IL-6, IL-8, and TNF-α in the colon was determined by qPCR. **(B)** Expression of secretory IgA (SIgA) in different segments of the intestine was assessed through immunohistochemistry. **(C)** The fold change of IFN-β, IFN-γ, ISG15, and OAS1 in the colon was determined by qPCR. Data are presented as mean ± SD (*n* = 3). *p < 0.05, **p < 0.01, ***p < 0.001, ****p < 0.0001.

To further explore the protective effects of PRV XJ Del gE/gI/TK on the intestine of piglets, we examined and compared the mRNA levels of inflammatory and antiviral factors among different groups. The mRNA levels of IL-1β, IL-10, and TNF-α in the vaccination group were significantly decreased compared to the vehicle group. Furthermore, there were no significant differences in IL-1β and TNF-α mRNA levels between the vaccination and mock groups. The mRNA levels of IL-6 and IL-8 did not show significant differences among all groups. On the other hand, the antiviral factors IFN-β, IFN-γ, ISG15, and OAS1 were significantly upregulated in the vaccination group compared to the vehicle group. Additionally, both the vaccination and vehicle groups exhibited significantly higher levels of antiviral factors compared to the mock group (Figure 7A). SIgA is a crucial immunoglobulin in mucosal immunity. Therefore, we also assessed the presence of SIgA-secreting cells in each intestinal segment using IHC. The results demonstrated that the proportion of SIgA-positive cells in all intestinal segments, except the duodenum, was higher in the vaccination group compared to the mock group and the vehicle group (Figure 7B). In summary, the vaccination group exhibited inhibited inflammation and strong activation of intestinal immunity.

## 4 Discussion

Several studies have demonstrated the efficacy of PRV gene-deleted vaccines in protecting piglets from variant PRV strains and preventing neurological and respiratory symptoms (Xu et al., 2022; Jiang et al., 2023). However, little research has focused on the effects of PRV on the gut and whether gene-deleted vaccines can counteract these negative effects. Existing research on PRV and its impact on the intestinal tract has indicated negative effects, such as inflammation and microbiota disruption (Ezura et al., 1995). It has been suggested that intestinal damage may be one of the hallmark injuries caused by PRV. Therefore, intestinal damage should also be considered as one of the evaluation indicators for PRV vaccines. In this study, we aimed to investigate the protective effects of a PRV vaccine, based on a previously constructed PRV strain with deletions in the gE/gI/TK genes, specifically analyzing its impact on the intestinal tract. The results of our study indicate that PRV infection significantly affects the intestinal condition of piglets. The PRV XJ Del gE/gI/TK vaccine effectively protects against intestinal damage, making it a promising candidate for the prevention and control of PRV, particularly in the context of the intestinal tract.

It is widely recognized that PRV can induce diarrhea in piglets, although the exact mechanisms underlying this phenomenon remain unclear (Zhang et al., 2019; Sehl and Teifke, 2020; Tan et al., 2021). Our data showed that not all PRV-infected piglets developed diarrhea, as only one out of three piglets in the vehicle group exhibited diarrhea symptoms. Interestingly, previous findings by Zhang et al. (2019) demonstrated the absence of viral load in the gut of piglets following intranasal PRV infection. However, we observed high viral loads of PRV in multiple segments of the intestine, particularly in the colon. This discrepancy may be attributed to the use of piglets of different ages, with older pigs possessing more mature immune systems capable of resisting PRV infection (Verpoest et al., 2017). While diarrhea may not manifest in all piglets, it appears that intestinal damage is a common consequence of PRV infection. Previous studies have reported intestinal damage caused by PRV and other herpesvirus subfamilies, such as HSV-1 (Narita et al., 1984; Ezura et al., 1995; Khoury-Hanold et al., 2016). In comparision to the previously reported necrosis and inflammatory infiltration, we observed atrophy of intestinal villi and hyperplasia of crypts mainly in the duodenum, jejunum, and ileum, indicating compromised absorption and digestion capacity in the small intestine (Narita et al., 1984; Ezura et al., 1995; Zhang et al., 2019). In the colon of the vehicle group, we observed a reduction in mucus content and the presence of edema. AB-PAS staining of the colon further confirmed a significant decrease in goblet cells secreting mucus in the vehicle group. Intriguingly, the ileum exhibited the lowest viral load, while the colon had the highest viral load. Hence, we speculate that the severe pathological injury observed in the ileum and colon may be influenced not only by viral replication but also by intestinal flora regulation. Compared to the duodenum and jejunum, the ileum and colon harbor a more diverse flora composition and a larger number of bacteria (Mowat and Agace, 2014). Consequently, an imbalance in the intestinal flora is more likely to disrupt intestinal homeostasis. Previous reports have indicated the detrimental effects of PRV infection on the intestinal microbial community, characterized by a decrease in the abundance of beneficial bacteria, including *Lactobacillus* species, and an increase in several potential pathogens (Zhang et al., 2019). *Lactobacillus* is known to contribute to the establishment of intestinal homeostasis by inhibiting pathogen colonization, alleviating viral infection, and producing antibacterial substances (Maragkoudakis et al., 2010; Hou et al., 2015). Overall, these findings underscore the negative effects of PRV infection on the intestine of piglets, involving intestinal damage and perturbations in the intestinal microbial community.

It has been well-documented that inflammation in the intestine can disrupt intestinal barrier function, leading to an increase in intestinal barrier permeability and a decrease in the expression of tight junction proteins (Xun et al., 2021). This phenomenon is observed in many gastrointestinal diseases, where secondary pathophysiological changes occur in the intestine following damage to the intestinal barrier (Ramos and Papadakis, 2019; Iyer and Corr, 2021). These changes often include intestinal edema, increased permeability of the intestine, and disruptions in the internal environment, which further contribute to the aggravation of intestinal damage. In our study, we observed that the levels of multiple inflammatory cytokines in the intestine were upregulated in the vehicle group after the PRV challenge. These cytokines include IL-1β, IL-6, IL-8, IL-10, and TNF-α. It is well-established that IL-1β and TNF-α are among the main pathogenic factors that contribute to damage in conditions such as Crohn’s disease and other inflammatory bowel diseases. IL-1β and TNF-α can exert their effects by reshaping cytoskeletal microfilaments through the MLCK-MLC and Rho A-ROCK-MLC signaling pathways (Abraham et al., 2022; Meyer et al., 2023). This leads to the contraction and increased tonicity of the actomyosin ring, ultimately causing the redistribution and destruction of tight junction proteins. To further investigate the effects of PRV on the intestinal barrier, we measured the concentration of DAO in plasma using an ELISA assay, and we examined the expression of tight junction proteins (occludin and zo-1) in the colon using Western blotting. Compared to the mock group and vaccination group, we observed an increase in plasma DAO concentration and a significant depletion of occludin and zo-1 expression in the colon of the vehicle group following the PRV challenge. This suggests that PRV may manipulate the breakdown of the intestinal barrier through inflammatory cytokines. In contrast, the vaccination group exhibited significantly reduced levels of inflammatory cytokines, and no increase in plasma DAO concentration was observed. Based on these findings, we hypothesize that the PRV XJ delgE/gI/TK vaccine protects intestinal barrier function from damage by inhibiting inflammation.

During viral infections, both epithelial cells and innate immune cells detect the presence of viral destruction through pattern recognition receptors located on the cytoplasm and cell membrane (Marques and Boneca, 2011). This recognition triggers a cascade of intracellular signaling pathways, leading to the activation of various host innate immune factors, including type I and type III interferons (Takeuchi and Akira, 2010). These interferons, in turn, induce the expression of multiple interferon-stimulated genes. However, PRV has developed various mechanisms to inhibit interferon production and evade active antiviral innate immunity (Ye et al., 2022). In our study, we observed significantly increased mRNA levels of IFN-β, IFN-γ, ISG15, and OAS1 in the gut of the vaccination group compared to the vehicle group. This may be attributed to the deletion of the gE, gI, and TK genes in PRV, which potentially eliminates its ability to evade host innate immunity (Pomeranz et al., 2005; Ye et al., 2022). As a result, the PRV variant is more capable of activating the expression of antiviral factors in the host. SIgA is a crucial effector of mucosal immunity and plays a significant role in the host’s primary defense against pathogenic microorganisms (Pietrzak et al., 2020). Therefore, we examined the differences in SIgA expression among the three groups. Our results demonstrated that the proportion of SIgA-positive cells in all intestinal segments, except for the duodenum, was significantly higher in the vaccination group compared to the vehicle group. The unique result in the duodenum may be attributed to the distribution pattern of IgA plasma cells along the intestinal segment. The number of IgA-producing plasma cells gradually increases from the duodenum to the colon, resulting in a distinct pattern of SIgA expression (Mowat and Agace, 2014).

## Data availability statement

The original contributions presented in the study are included in the article/supplementary material, further inquiries can be directed to the corresponding authors.

## Ethics statement

The animal study was approved by All experimental procedures were reviewed and approved by the Sichuan Agriculture University Animal Care and Use Committee (license number SCXK [Sichuan] 2019–187). The study was conducted in accordance with the local legislation and institutional requirements.

## Author contributions

YZ: Conceptualization, Formal analysis, Methodology, Software, Validation, Visualization, Writing – original draft, Writing – review & editing. LX: Software, Writing – review & editing. QT: Writing – review & editing, Software. ZL: Writing – review & editing, Formal analysis. JW: Writing – review & editing, Methodology. TX: Writing – review & editing, Formal analysis. SL: Project administration, Writing – review & editing. YA: Project administration, Writing – review & editing. ZX: Resources, Writing – review & editing. LZ: Resources, Supervision, Writing – review & editing, Conceptualization.
